# Enzymatic Oxidation of Aflatoxin M_1_ in Milk Using CotA Laccase

**DOI:** 10.3390/foods13223702

**Published:** 2024-11-20

**Authors:** Yongpeng Guo, Hao Lv, Zhiyong Rao, Zhixiang Wang, Wei Zhang, Yu Tang, Lihong Zhao

**Affiliations:** 1 College of Animal Science and Technology, Henan Agricultural University, Zhengzhou 450046, China; guoyp@henau.edu.cn (Y.G.); lcxhau@163.com (H.L.); rzyhau@163.com (Z.R.); weizhang@henau.edu.cn (W.Z.); 2 College of Animal Science and Technology, China Agricultural University, Beijing 100193, China; m17801115235@163.com

**Keywords:** aflatoxin M_1_, CotA laccase, oxidation, milk

## Abstract

Aflatoxin M_1_ (AFM_1_) in milk poses a significant threat to human health. This study examined the capacity of *Bacillus licheniformis* CotA laccase to oxidize AFM_1_. The optimal conditions for the CotA laccase-catalyzed AFM_1_ oxidation were observed at pH 8.0 and 70 °C, achieving an AFM_1_ oxidation rate of 86% in 30 min. The *K*m and Vmax values for CotA laccase with respect to AFM_1_ were 18.91 μg mL^−1^ and 9.968 μg min^−1^ mg^−1^, respectively. Computational analysis suggested that AFM_1_ interacted with CotA laccase via hydrogen bonding and van der Waals interactions. Moreover, the oxidation products of AFM_1_ mediated by CotA laccase were identified as the C3-hydroxy derivatives of AFM_1_ by HPLC-FLD and UPLC-TOF/MS. Toxicological assessment revealed that the hepatotoxicity of AFM_1_ was substantially reduced following oxidation by CotA laccase. The efficacy of CotA laccase in removing AFM_1_ in milk was further tested, and the result showed that the enzyme agent achieved an AFM_1_ removal rate of 83.5% in skim milk and 65.1% in whole milk. These findings suggested that CotA laccase was a novel AFM_1_ oxidase capable of eliminating AFM_1_ in milk. More effort is still needed to improve the AFM_1_ oxidase activity of CotA laccase in order to shorten the processing time when applying the enzyme in the milk industry.

## 1. Introduction

Milk is commonly recognized as a fundamental food product that provides a comprehensive range of readily accessible and bioavailable nutrients crucial for human growth, development, and health maintenance. Moreover, the consumption of milk products is correlated with a decreased risk of cardiovascular diseases, notably stroke [1]. However, the food hygiene of milk products is often compromised by aflatoxin M_1_ (AFM_1_), which has been a great concern for consumers. AFM_1_, known as a milk toxin, is the hydroxylated form of aflatoxin B_1_ (AFB_1_), which is generated by the hepatic mitochondrial cytochrome P450 enzymes and excreted in the milk, feces, and urine of lactating animals after the consumption of an AFB_1_-contaminated diet. AFM_1_ appears in dairy cow milk within 12 h of the first AFB_1_ administration and clears from milk after a 72 h AFB_1_ withdrawal period [2,3]. The carry-over rate of dietary AFB_1_ to milk AFM_1_ in dairy cattle ranges between 0.3 and 6.2% depending on the health conditions, milk yield, feed type, and level of contamination [4]. AFM_1_ remains stable during various milk processing methods, including sterilization, pasteurization, and fermentation, and its presence has been documented throughout the entire dairy supply chain, encompassing products such as milk powder, cheese, and yogurt [5]. The prevalence of AFM_1_ in milk poses significant health risks, particularly for vulnerable populations such as infants and the elderly, who may be more susceptible to its harmful effects. Although AFM_1_ has been found to be 10-fold less carcinogenic than AFB_1_, IARC continues to classify it as a class 1 carcinogen [6]. Given the potential health risks of AFM_1_, over 60 countries have developed guidelines for the maximum residue level of milk AFM_1_. Among these, 34 countries, including China, have set the limit at 0.5 µg L^−1^ in fluid milk. In contrast, the European Union has adopted a more stringent maximum level at 0.05 µg L^−1^ for AFM_1_ in raw milk and milk intended for the production of dairy products.

The development of efficient and environmentally sustainable methods to reduce AFM_1_ contamination in milk is urgently needed. Milk contamination can be reduced either indirectly, by preventing dairy animals’ dietary exposure to AFB_1_, or directly, by eliminating AFM_1_ from contaminated milk. The implementation of good agricultural practices (GAPs), such as crop rotation, soil management, insect damage control, the selection of fungal-resistant crop varieties, and timely harvesting, is helpful for the inhibition of mold infection and AFB_1_ generation [7]. Unfortunately, these pre-harvest prevention strategies are not consistently sufficient to produce AFB_1_-free crops. To mitigate the carry-over of feed-derived AFB_1_ to milk AFM_1_, dairy farming commonly employs organic and inorganic adsorbents like yeast cell wall extracts [8], calcium montmorillonite clay [9], and hydrated sodium calcium aluminosilicates [10], which can bind AFB_1_ within the gastrointestinal tract of animals. Moreover, several reports documenting the capacity of yeasts and lactic acid bacteria to bind AFM_1_ in contaminated milk are available [11,12,13]. Despite favorable outcomes of microbial cells in milk AFM_1_ elimination, the commercialization of adsorption-associated technologies still has limitations, like the instability of microbial cell–AFM_1_ complexes, the increase in microbial loads, and the loss of nutritional value [14].

In recent years, research emphasis has gradually shifted toward the microbial and enzymatic degradation of aflatoxins in food commodities, which transforms aflatoxins into less harmful metabolites, while preserving the palatability and nutritional value of food and feed. A number of AFB_1_-degrading fungal and bacterial strains have been identified, including *Pleurotus eryngii* [15], *Ganoderma sinense* [16], *Myroides odoratimimus* [17], *Pseudomonas aeruginosa* [18], *Cellulosimicrobium funkei* [19], and *Bacillus licheniformis* [20]. Several studies have also documented that AFB_1_ can be degraded by laccase [21,22], peroxidase [23,24], dipeptidyl peptidase [25], and F_420_H_2_-dependent reductase [26]. However, the capacity and application potential of these microorganisms and enzymes for degrading AFM_1_ have been rarely studied. In this study, we expanded upon previous findings that demonstrated the capacity of CotA laccase to directly oxidize AFB_1_ without the need for redox mediators, resulting in the formation of aflatoxin Q_1_ and epi-aflatoxin Q_1_ [22]. We further investigated the enzymatic characteristics of CotA laccase in oxidizing AFM_1_. CotA laccase-mediated AFM_1_ oxidation products were identified. Additionally, we evaluated the toxicity of AFM_1_ oxidation products using hepatocytes L-02. The interaction between CotA laccase and AFM_1_ was investigated by molecular docking simulation. Furthermore, the efficacy of CotA laccase in removing AFM_1_ in both skim milk and whole milk was evaluated for the first time. This work is expected to contribute to the advancement of enzyme-based strategies to mitigate AFM_1_ contamination in milk products.

## 2. Materials and Methods

### 2.1. Materials and Reagents

CotA laccase was expressed and purified from *Escherichia coli* Rossta (DE3) bearing recombinant expression vector pET31b-CotA as described previously [22]. AFM_1_ was purchased from Sigma-Aldrich (Shanghai, China). AFM_1_ immune-affinity columns were obtained from Clover Technology Group, Inc. (Beijing, China). Ultrahigh-temperature-treated (UHT) whole milk and skim milk were purchased from a local supermarket.

### 2.2. Enzymatic Characteristics of CotA Laccase for Oxidizing AFM_1_

The enzymatic characteristics of CotA laccase in the oxidation of AFM_1_ were characterized. To assess the impact of pH on AFM_1_ oxidation, a reaction mixture comprising 1 μg mL^−1^ of AFM_1_ and 0.1 U mL^−1^ of CotA laccase was incubated at 37 °C across a range of pH conditions (pH 4.0 to 9.0) for 12 h. The impact of temperature on AFM_1_ oxidation was assessed by incubating 1 μg mL^−1^ of AFM_1_ with 0.1 U mL^−1^ of CotA laccase across a temperature range of 30 to 80 °C for 30 min. Additionally, the influence of metal ions on CotA laccase-mediated AFM_1_ oxidation was evaluated by pre-incubating 0.5 U mL^−1^ of CotA laccase with 10 mM of different metal ions at 37 °C and pH 7.0 for 10 min. Subsequently, AFM_1_ (1 μg mL^−1^) was introduced, and the mixture was incubated at 37 °C for an additional 30 min. The Michaelis–Menten kinetics of CotA laccase-mediated AFM_1_ oxidation was analyzed at 37 °C and pH 7.0. Initial reaction velocities were determined by quantifying the reduction in AFM_1_ concentration in 30 min following the introduction of 0.1 U mL^−1^ of CotA laccase. The experimental data were fitted to the Michaelis–Menten equation to derive the *K*_m_ and V_max_ values with Graphpad Prism 7.0.

### 2.3. AFM_1_ Concentration Determination by HPLC

Chromatographic analysis was conducted utilizing a high-performance liquid chromatography (HPLC) system (Shimadzu LC-10 AT, Shimadzu, Tokyo, Japan), which was equipped with a post-column photochemical derivatization unit and an RF-20A fluorescence detector (Shimadzu, Tokyo, Japan). The separation process employed an isocratic method using a reverse-phase column (DIKMA, C18, 5 μm, 150 × 4.6 mm). The mobile phase consisted of acetonitrile, methanol, and water in a volumetric ratio of 24:8:68. The excitation and emission wavelengths were set at 365 nm and 435 nm, respectively. The flow rate was maintained at 1.0 mL min^−1^, and the injection volume was 20 μL.

### 2.4. UPLC-TOF/MS Analysis of CotA Laccase-Mediated AFM_1_ Oxidation Products

UPLC-TOF/MS was applied to identify CotA laccase-mediated AFM_1_ oxidation products. Chromatographic separation was performed using an Acquity UPLC BEH C18 column (1.7 μm, 2.1 × 100 mm) (Waters, Milford, MA, USA). The mobile phase, delivered at a flow rate of 0.3 mL min^−1^, comprised a binary solvent system of water and methanol. The elution gradient was as follows: 0–10 min, 5% to 50% methanol; 10–11 min, 50% to 95% methanol; 11–13 min, 95% methanol; and 13–15 min, 95% to 5% methanol. The sample injection volume was set at 2 μL. Mass spectral data were acquired using a Waters Xevo G2-XS QTOF mass spectrometer (Waters, Milford, MA, USA). The TOF-MS was conducted in ESI positive mode. The mass spectrometry parameters were set as follows: ion source temperature at 350 °C, nitrogen gas flow rate at 10 L min^−1^, capillary voltage at 3000 V, and scan range from *m*/*z* 50 to 1000.

### 2.5. Homology Modeling and Molecular Docking

The three-dimensional structure of *B. licheniformis* ANSB821 CotA laccase was constructed with the X-ray crystal structure of *B. subtilis* 168 CotA laccase (PDB entry 2WSD) [27] as a template with the SWISS-MODEL sever. The three-dimensional structure of AFM_1_ was generated using ChemBioDraw 2014. Molecular docking was conducted using MOE Dock in MOE v2014.0901. The docking procedure adhered to the “induced fit” protocol, which permitted the side chains of the receptor pocket to adjust in response to ligand conformations, while maintaining a positional constraint.

### 2.6. Cytotoxicity Evaluation of AFM_1_ Oxidation Products

Human fetal hepatocyte cell line L-02 obtained from Tongpai Biotechnology Co., Ltd. (Shanghai, China) was cultured in Dulbecco’s Modified Eagle’s Medium (DMEM) containing 10% fetal bovine serum and 100 U mL^−1^ of penicillin and streptomycin. Cell viability was assessed using a CCK-8 assay kit (Biosharp, Beijing, China). L-02 cells were inoculated in a 96-well plate (1 × 10^4^ cells each well), and treated with 100 μM of AFM_1_ and CotA laccase-catalyzed AFM_1_ oxidation products for 24 h. Afterwards, 10 μL of CCK-8 reagent was introduced into each well, followed by incubation at 37 °C for 1 h. The absorbance value at 450 nm was taken with a microplate reader to assess cell viability. The extracellular lactate dehydrogenase (LDH) activity and cell apoptosis rate of L-02 cells were also measured after exposure to 100 μM of AFM_1_ and CotA laccase-catalyzed AFM_1_ oxidation products for 24 h. LDH activity was determined using a commercially available kit (Biosharp, Beijing, China). The rate of cellular apoptosis was assessed utilizing the Annexin V-fluorescein isothiocyanate (FITC) Apoptosis Detection Kit (Uelandy, Suzhou, China).

### 2.7. Performance of CotA Laccase in Degrading AFM_1_ in Milk

The experiments regarding the elimination of AFM_1_ in whole milk and skim milk by CotA laccase were carried out in 100 mL conical flasks. Milk samples were spiked with AFM_1_ at a concentration of 2.0 ng mL^−1^. To study the effect of the amount of CotA laccase on AFM_1_ oxidation, CotA laccase was added to 20 mL of spiked milk to reach a final concentration ranging from 0.1 to 2.0 U mL^−1^. The control was prepared with sodium phosphate buffer in place of CotA laccase. The samples were incubated at 37 °C for 12 h in a water bath. The effect of incubation time on AFM_1_ oxidation was determined at 3, 6, 12, 18, and 24 h, respectively, with 1.0 U mL^−1^ of CotA laccase at 37 °C. All experiments were repeated three times. The concentration of AFM_1_ in milk was measured with HPLC. Briefly, the milk samples were centrifuged at 6000 rpm for 20 min. Then, 10 mL of supernatant was collected and applied to an AFM_1_ immune-affinity column at a steady flow rate of 1 mL min^−1^. The column was washed twice with 10 mL of ultrapure water. AFM_1_ was eluted from the column with 2 mL of methanol. The eluent was evaporated to dryness at 40 °C under a N_2_ stream and reconstituted in 1 mL of ultrapure water before loading in the HPLC system. Validation parameters for the determination of AFM_1_ concentration in milk with the HPLC-FLD method are summarized in Table S1.

## 3. Results and Discussion

### 3.1. Enzymatic Properties of CotA Laccase for Oxidizing AFM_1_

Laccases, a class of multicopper oxidases, possess the ability to oxidize a diverse array of aromatic compounds [28]. Due to their versatility, stability, and broad substrate specificity, numerous applications of laccases have been developed within the food industry over recent decades, including roles in baking, fruit juice clarification, wine and beer stabilization, and sugar beet pectin gelation [29]. Our previous study found that CotA laccase could serve as a novel aflatoxin oxidase [22].

**Figure 1:**
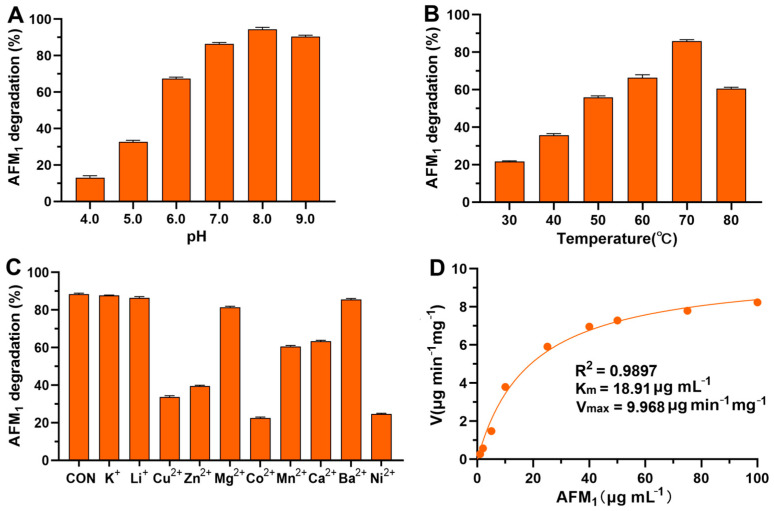
Enzymatic properties and kinetics of CotA laccase-mediated AFM_1_ oxidation. Effect of pH (**A**), temperature (**B**), and metal ions (**C**) on CotA laccase-mediated AFM_1_ oxidation. (**D**) Michaelis–Menten plot of CotA laccase-catalyzed AFM_1_ oxidation.

In the current study, we further characterized the enzymatic properties and kinetics of CotA laccase in the oxidation of AFM_1_. The influence of pH, temperature, and metal ions on CotA laccase-catalyzed AFM_1_ oxidation was initially examined. The optimal pH range for CotA laccase in oxidizing AFM_1_ was observed at pH 7.0 to 9.0, with the oxidation rate remaining above 86%, and the highest AFM_1_ oxidation rate of 94% was obtained at pH 8.0 (Figure 1A). The substrate-dependent optimal pH is a characteristic feature of laccases. Previously, the optimal pH values for *B. licheniformis* CotA laccase in the oxidation of 2, 2′-Azino-bis-(3-ethylbenzothiazoline-6-sulphonic acid) (ABTS), syringaldazine, 2,6-dimethoxyphenol, and AFB_1_ were determined to be 4.2, 6.8, 7.2, and 8.0, respectively [22]. Therefore, it is essential to determine the optimal pH for the oxidation of various substrates using CotA laccase. The effect of temperature on the oxidation of AFM_1_ by CotA laccase is illustrated in Figure 1B. The oxidation rate of AFM_1_ increased from 22% to 86% as the temperature rose from 30 °C to 70 °C, and then dropped to 60% at 80 °C. This observation aligns with the known characteristics of *Bacillus* CotA laccases, which are recognized as thermophilic enzymes with optimal activity within a temperature range of 50 °C to 70 °C [30]. The influence of various metal ions on the CotA laccase-mediated oxidation of AFM_1_ was examined, as depicted in Figure 1C. The presence of K^+^, Li^+^, and Ba^2+^ exhibited minimal impact on AFM_1_ oxidation by CotA laccase, whereas Mn^2+^ and Ca^2+^ at 10 mM caused a more than 15% reduction in the AFM_1_ oxidation rate. Moreover, Cu^2+^, Zn^2+^, Co^2+^, and Ni^2+^ could strongly inhibit the AFM_1_-oxidizing activity of CotA laccase. Indeed, the inhibitory effects of Cu^2+^, Zn^2+^, Co^2+^, and Ni^2+^ are commonly observed in laccases, potentially due to their interaction with the electron transport system of the enzymes [31,32].

The kinetic parameters of CotA laccase with respect to AFM_1_ were determined by fitting the experimental data to the Michaelis–Menten plot (Figure 1D). The coefficient of determination (R^2^) was 0.9897, indicating an excellent fit of the model to the data. The *K*_m_ and V_max_ values were calculated to be 18.91 μg mL^−1^ and 9.968 μg min^−1^ mg^−1^, respectively. This strong correlation of the Michaelis–Menten model further implies that AFM_1_ is a novel substrate of CotA laccase.

### 3.2. Identification of CotA Laccase-Mediated AFM_1_ Oxidation Products

**Figure 2:**
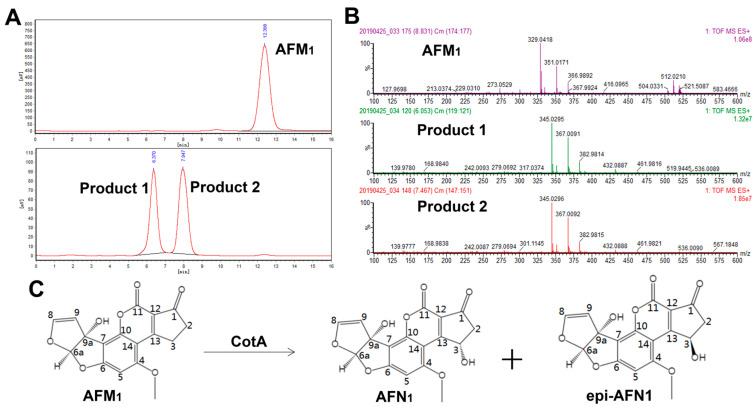
Identification of CotA laccase-mediated AFM_1_ oxidation products. (**A**) HPLC chromatograms of AFM_1_ and CotA laccase-mediated AFM_1_ oxidation products. (**B**) Mass spectra analysis of AFM_1_ and CotA laccase-mediated AFM_1_ oxidation products. (**C**) The reaction scheme for AFM_1_ oxidation by CotA laccase.

CotA laccase-mediated AFM_1_ oxidation products in sodium phosphate buffer were characterized by HPLC-FLD and UPLC-TOF/MS analysis. The oxidation of AFM_1_ resulted in the formation of two products with retention times earlier than that of AFM_1_ (Figure 2A). The oxidation products were further characterized by MS analysis, as shown in Figure 2B and Figure S1. The two products showed the same molecular ion peak at *m*/*z* 345.03 ([M + H]^+^), *m*/*z* 367.01 ([M + Na]^+^) and *m*/*z* 382.98 ([M + K]^+^), corresponding to the formula of C_17_H_12_O_8_, which suggested the addition of a single oxygen to AFM_1_ (C_17_H_12_O_7_). Aflatoxins can be degraded by several mechanisms, such as epoxidation, hydroxylation, dehydrogenation, and reduction. Research by Loi et al. [21] suggested that treating aflatoxins with Lac2 laccase led to cleavage of the lactone ring, whereas Alberts et al. [33] found that pure laccase of *T. versicolor* changed the double bond of the furan ring. Moreover, Liu et al. [34] reported that both the furan ring and lactone ring were abolished after treatment by *Bacillus pumilus* superoxide dismutase.

Our previous study confirmed that CotA laccase catalyzed the C3-hydroxylation of AFB_1_, leading to the formation of a pair of epimers, AFQ_1_ and epi-AFQ_1_ [22]. Based on HPLC-FLD and UPLC-TOF/MS analysis in this study, the same degradation pathway was proposed for AFM_1_, as shown in Figure 2C. The two transformation products were named aflatoxin N_1_ (AFN_1_, 3*S*) and epi-aflatoxin N_1_ (epi-AFN_1_, 3*R*). Further study is necessary to confirm their chemical structures using nuclear magnetic resonance spectroscopy.

### 3.3. Interaction of AFM_1_ with CotA Laccase by Molecular Docking

**Figure 3:**
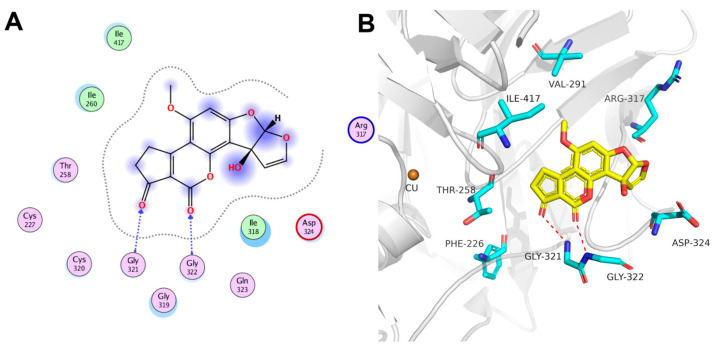
Molecular docking analysis of AFM_1_ with CotA laccase. (**A**) The two-dimensional interaction model of AFM_1_ with CotA laccase. (**B**) The three-dimensional interaction model of AFM_1_ with CotA laccase.

A docking simulation study was conducted to examine the interaction of AFM_1_ with CotA laccase. The docking score for the binding mode of AFM_1_ with CotA laccase was calculated to be −5.50 kcal mol^−1^ (Figure 3). The binding free energy was negative, reflecting that AFM_1_ oxidation by CotA laccase was spontaneous. The main interaction observed between AFM_1_ and CotA laccase was hydrogen bonding. Specifically, the two carbonyl oxygen atoms of the lactone group in AFM_1_, acting as hydrogen bond acceptors, formed hydrogen bonds with the backbone of Gly321 and Gly322, respectively. Additionally, AFM_1_ provided good van der Waals interaction with Ile260, Ile318, Cys320, and Ile417. Liu et al. [35] also reported that hydrogen bonding was the primary driving force for the binding between aflatoxins (AFB_1_, AFB_2_, AFG_1_, and AFG_2_) and *Trametes* sp. C30 laccase. The in silico investigation conducted by Dellafiora et al. [36] indicated distinct binding configurations of AFB_1_ and AFM_1_ within the catalytic sites of laccases derived from *Trametes versicolor*. The hydroxyl group on the difuran moiety of AFM_1_ enabled a more profound penetration into the catalytic pocket of *T. versicolor* laccases, attributed to the establishment of an additional hydrogen bond within the AFM_1_–laccase complex. However, we found that the simulated binding model of AFM_1_ to CotA laccase was the same as the AFB_1_-CotA interaction model [22], which was in agreement with the finding in this study that AFB_1_ and AFM_1_ underwent same molecular modification by CotA laccase.

### 3.4. Hepatotoxicity Evaluation of AFM_1_ Oxidation Products

**Figure 4:**
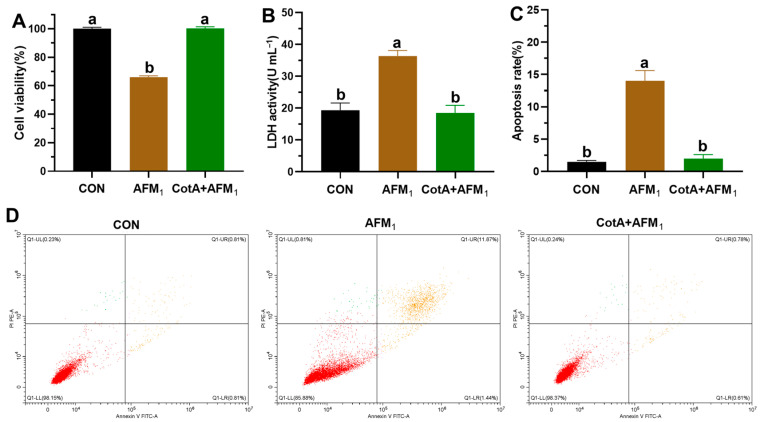
Evaluation of the cytotoxic effects of AFM_1_ and its oxidation products. (**A**) Viability of L-02 cells following exposure to 100 μΜ of AFM_1_ and CotA laccase-catalyzed AFM_1_ oxidation products. (**B**) LDH activity. (**C**,**D**) Apoptosis rate of L-02 cells. Different letters denote statistically significant differences between groups (*p* < 0.05).

The liver, being the main organ for mycotoxin metabolism, is primarily impacted by AFM_1_ toxicity. A previous study has documented that AFM_1_ exposure results in liver damage, characterized by poor cytoplasmic integrity, inflammatory infiltration, sinusoidal dilation, and nuclear irregularities [37]. Thus, this study assessed the cytotoxicity of AFM_1_ oxidation products using hepatocytes L-02. As shown in Figure 4A, the viability reduced remarkably following exposure to 100 μΜ of AFM_1_ for 24 h. Conversely, treatment with 100 μΜ of CotA laccase-mediated AFM_1_ oxidation products did not notably impact cell viability. The detrimental impact of AFM_1_ on L-02 cells was corroborated by a notable increase in the extracellular LDH activity of the AFM_1_ group (Figure 4B). LDH, a cytoplasmic enzyme, is rapidly released into the culture medium upon cytomembrane leakage, serving as an indicator of cell damage. Consistent with the result of cell viability, no significant difference was observed in extracellular LDH activity between the group treated with AFM_1_ oxidation products and the control group. Furthermore, flow cytometry analysis indicated a significant increase in cell apoptosis rate in the AFM_1_ group compared to the control, whereas the AFM_1_ oxidation products did not induce cell apoptosis (Figure 4C,D). Collectively, these findings suggest that the CotA laccase-mediated oxidation of AFM_1_ effectively detoxifies its hepatotoxic effects.

### 3.5. Performance of CotA Laccase to Degrade AFM_1_ in Milk

**Figure 5:**
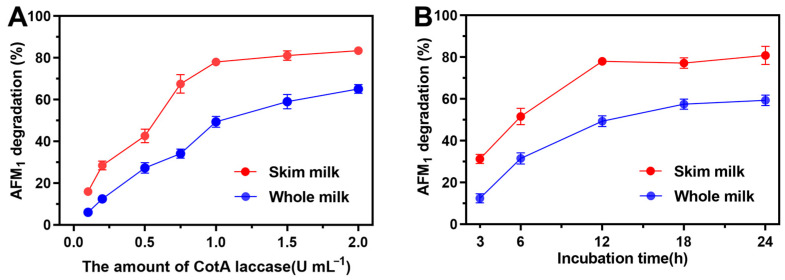
Elimination of AFM_1_ in milk by CotA laccase. Effect of CotA laccase amount (**A**) and incubation time (**B**) on AFM_1_ degradation rate in skim milk and whole milk.

The performance of CotA laccase to eliminate AFM_1_ in milk was further investigated. Whole milk and skim milk spiked with AFM_1_ at a concentration of 2 ng mL^−1^ were subjected to treatment with CotA laccase. The impact of the amount of CotA laccase on AFM_1_ elimination is shown in Figure 5A. The AFM_1_ removal percentage increased with the increase in CotA laccase from 0.1 to 2.0 U mL^−1^, and the maximum removal rates were 83.5% and 65.1% for skim milk and whole milk, respectively.

As summarized in Table 1, several research efforts have been dedicated to the enzymatic elimination of AFM_1_ in milk. Marimón Sibaja et al. [38] reported that commercial horseradish peroxidase (HRP) could reduce AFM_1_ content in UTH milk by 65.0%. Kerstner et al. [39] used crude extract of peroxidase from rice bran to eliminate AFM_1_ in milk and achieved the removal percentage of 71.2%. However, H_2_O_2_ was required for the oxidation of AFM_1_ by peroxidases. With respect to large-scale milk processing, CotA laccase has the advantage of directly oxidizing AFM_1_ by using O_2_ as an electron acceptor. The food matrix can exert a significant influence on the performance of enzymes in food processing. The AFM_1_ elimination efficiency of CotA laccase was significantly reduced by fat and fat-soluble vitamins in whole milk.

The impact of incubation time on the elimination of AFM_1_ is given in Figure 5B. The removal percentage of AFM_1_ in milk by CotA laccase increased with increasing incubation time, which reached 80.9% and 59.3% for skim milk and whole milk, respectively, at 37 °C after 24 h. Procedures possessing high applicable potential for the elimination of AFM_1_ in milk should meet a set of criteria including safety, rapidity, and selectivity [43]. The negative impact of CotA laccase treatment on the nutritive value, organoleptic profiles, and technological properties of milk should be further investigated.

## 4. Conclusions

This study presents the first characterization of the enzymatic properties and kinetics of CotA laccase in oxidizing AFM_1_. The optimal oxidation of AFM_1_ by CotA laccase was observed at pH 8.0 and 70 °C. Molecular docking simulations suggested that AFM_1_ could interact with CotA laccase via hydrogen bonding and van der Waals interactions. The CotA-AFM_1_ complex formed two hydrogen bonds via the carbonyl oxygen atoms and the Gly321 and Gly322 residues. Moreover, HPLC-FLD and UPLC-TOF/MS analysis indicated that CotA laccase oxidized AFM_1_ to its C3-hyhroxy derivatives AFN_1_ and epi-AFN_1_. Furthermore, the oxidation of AFM_1_ catalyzed by CotA laccase was observed to significantly reduce its cytotoxicity in hepatocytes. Subsequently, CotA laccase was employed to eliminate AFM_1_ in milk, with the removal percentage being influenced by factors such as milk type, CotA laccase concentration, and incubation duration. It was shown that 83.5% of AFM_1_ in skim milk and 65.1% of AFM_1_ in whole milk were eliminated by 2 U mL^−1^ of CotA laccase at 37 °C after 12 h. This study highlights the AFM_1_ oxidase activity of CotA laccase. Nonetheless, further research is necessary to increase the AFM_1_ removal efficiency of CotA laccase in milk by enzyme engineering technology.

## Figures and Tables

**Table 1:** Summary of enzymatic elimination of AFM_1_ in milk.

Enzyme	Origin	Reaction Conditions	Elimination Rate	Reference
HRP	*Amoracia rusticana*	0.015 U mL^−1^ HRP, 0.08% H_2_O_2_, 5 ng mL^−1^ AFM_1_, 30 °C for 8 h	65.0%	[38]
RBP	Rice bran	0.015 U mL^−1^ HRP, 0.08% H_2_O_2_, 5 ng mL^−1^ AFM_1_, 4 °C for 24 h	71.2%	[39]
SOD	*Bacillus pumilus*	1 U mL^−1^ SOD, 2 μg mL^−1^ AFM_1_, 40 °C for 24 h	26.0%	[34]
CAT	*Bacillus pumilus*	1 U mL^−1^ CAT, 2 μg mL^−1^ AFM_1_, 40 °C for 12 h	47.2%	[40]
POD1	*Bacillus pumilus*	1 U mL^−1^ POD1, 2 μg mL^−1^ AFM_1_, 35 °C for 12 h	22.4%	[41]
POD2	*Bacillus pumilus*	1 U mL^−1^ POD2, 2 μg mL^−1^ AFM_1_, 35 °C for 12 h	25.6%	
POD3	*Bacillus pumilus*	1 U mL^−1^ POD3, 2 μg mL^−1^ AFM_1_, 35 °C for 24 h	24.3%	
Lac	*Trametes versicolor*	20 mg mL^−1^ Lac, 0.5 ng mL^−1^ AFM1, 25 °C for 80 min	32.0%	[42]
CotA	*Bacillus licheniformis*	2 U mL^−1^ CotA, 2 ng mL^−1^ AFM_1_, 37 °C for 12 h	83.5% for skim milk; 65.1% for whole milk	This study

## Data Availability

The original contributions presented in the study are included in the article and Supplementary Materials; further inquiries can be directed to the corresponding authors.

## Supplementary Materials

The following supporting information can be downloaded at: https://www.mdpi.com/article/10.3390/foods13223702/s1, Figure S1: (**A**) Selected ion chromatography of AFM_1_; (**B**) Selected ion chromatography of CotA laccase-mediated AFM_1_ oxidation products. Table S1. Validation parameters for the determination of AFM_1_ concentration in milk with HPLC-FLD method.
