# Supplementary material for: Enzymatic Oxidation of Aflatoxin M1 in Milk Using CotA Laccase
Source: Foods. 2024 Nov 20;13(22):3702. doi: 10.3390/foods13223702 (PMC11593616; doi:10.3390/foods13223702)
Supplement: Supplementary file 1 [file foods-13-03702-s001.zip › foods-3300435-supplementary.pdf]

Table S1. Validation parameters for the determination of AFM_1 concentration in milk with HPLC-FLD method.

Item	LOD[1] [μg L^{-1}]	LOQ[2] [μg L^{-1}]	Recovery (%)	Repeatability (% RSD[3])
AFM1	0.005	0.015	93	5.1

[1], Limit of Detection; [2], Limit of Quantification; [3], Relative standard deviation

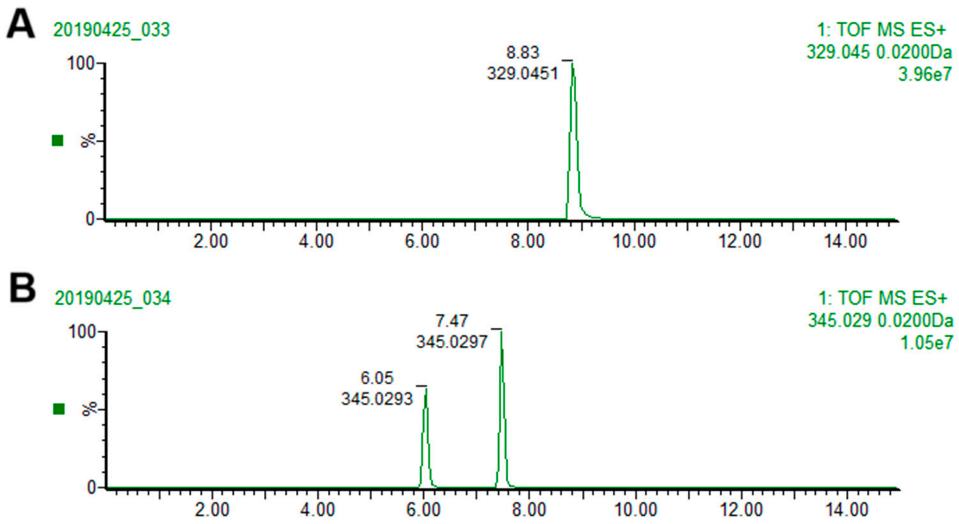

Figure S1. (**A**) Selected ion chromatography of AFM_1; (**B**) Selected ion chromatography of CotA laccase-mediated AFM_1 oxidation products.
